# Swarming of *P. aeruginosa*: Through the lens of biophysics

**DOI:** 10.1063/5.0128140

**Published:** 2023-09-28

**Authors:** Jean-Louis Bru, Summer J. Kasallis, Quantum Zhuo, Nina Molin Høyland-Kroghsbo, Albert Siryaporn

**Affiliations:** 1 Department of Molecular Biology and Biochemistry, University of California Irvine, Irvine, California 92697, USA; 2 Department of Physics and Astronomy, University of California Irvine, Irvine, California 92697, USA; 3 Department of Plant and Environmental Sciences, University of Copenhagen, Frederiksberg, Denmark

## Abstract

Swarming is a collective flagella-dependent movement of bacteria across a surface that is observed across many species of bacteria. Due to the prevalence and diversity of this motility modality, multiple models of swarming have been proposed, but a consensus on a general mechanism for swarming is still lacking. Here, we focus on swarming by *Pseudomonas aeruginosa* due to the abundance of experimental data and multiple models for this species, including interpretations that are rooted in biology and biophysics. In this review, we address three outstanding questions about *P. aeruginosa* swarming: what drives the outward expansion of a swarm, what causes the formation of dendritic patterns (tendrils), and what are the roles of flagella? We review models that propose biologically active mechanisms including surfactant sensing as well as fluid mechanics-based models that consider swarms as thin liquid films. Finally, we reconcile recent observations of *P. aeruginosa* swarms with early definitions of swarming. This analysis suggests that mechanisms associated with sliding motility have a critical role in *P. aeruginosa* swarm formation.

## INTRODUCTION

Swarming is a flagella-dependent form of collective movement in which a cell population expands on a semi-solid surface. This type of movement is observed across multiple bacterial phyla with genera including *Aeromonas*, *Bacillus*, *Escherichia*, *Proteus*, *Pseudomonas*, *Salmonella*, *Serratia*, *Vibrio*, and *Yersinia.*^1–9^ Although many Gram-positive and Gram-negative bacteria are capable of swarming, each species may require distinct environmental conditions to swarm or harbor specific biological features that impact swarm development. In laboratory settings, swarming is dependent on a number of environmental factors including nutrient availability and humidity.^10–14^ Different species exhibit diverse swarm patterns, which suggests that swarming is dependent on the unique details of their motility mechanisms.^15,16^ On the other hand, it is possible that distinct mechanisms among different species give rise to similar movements that have been classified together as swarms. It is, therefore, challenging to discuss a single model of swarming that encompasses all bacterial species. In this review, we focus on swarming in *P. aeruginosa* due to the abundance of experimental and modeling-based literature on swarming of this species. *P. aeruginosa* swarms are characterized by the outward expansion of the population from the original inoculation area and the formation of dendritic patterns, which are referred to here as tendrils. A growing consensus identifies two activities that are essential for this species to form such patterns: the production of surfactant and the activity of flagella.^5,17^ Multiple biological and biophysical studies have proposed different mechanisms by which surfactants and flagella could facilitate swarming. We aim to reconcile these interpretations and place them into context to provide a comprehensive understanding of swarming in *P. aeruginosa*.

Several swarming models have focused on the role of fluid mechanics in driving swarm expansion and in the formation of the dendritic patterns. These models invoke processes that are not widely considered by biology, including Marangoni flow, Darcy's law, and Saffman–Taylor instabilities.^18–20^ The connection between fluid mechanics-based models and the biological interpretations of swarming seems tenuous at first. Furthermore, it is unclear to what extent these models are complementary or mutually exclusive. However, models from both approaches are rooted in shared concepts, in particular, surface tension, osmolarity, and viscosity. Here, we review the relative contribution of biological and biophysical mechanisms to swarming. We find that both biological and fluid mechanics-based mechanisms need to be considered in order to explain the observations of *P. aeruginosa* swarming. We address the central question: what mechanisms in *P. aeruginosa* cause swarms to expand and form dendritic patterns? To achieve this end, we provide a background that summarizes the roles of rhamnolipids (RLs), quorum sensing (QS), and flagella in swarming and highlights knowledge gaps. We then focus on understanding how swarms expand and how tendrils develop, first reviewing the role of chemotaxis and how the sensing of QS-associated products could impact tendril development. We then consider the role of fluid mechanics-based mechanisms, including Marangoni flow, pressure-driven flow, and Darcy's law. In particular, the influx of fluid into the swarm has a critical role in swarm expansion and tendril development. We consider how these mechanisms explain recent observations of swarm development in which tendril movement can be altered by changing the flow of surfactant. This discussion aims to provide a unified biophysical interpretation of swarming in *P. aeruginosa*. Based on this interpretation, the Henrichsen definition of swarming is revisited, which raises the possibility that *P. aeruginosa* swarming involves sliding motility. Quantitative metrics of swarming are proposed to improve assessments and classifications of *P. aeruginosa* surface motility. We conclude with discussing the role of flagella in swarms, which raises important issues that should be addressed in future work.

## BACKGROUND

### Rhamnolipids decrease surface tension and surface friction

RLs are glycolipids that *P. aeruginosa* produces in relatively high abundance and that are critical for swarming motility. RLs are produced as a mixture of two forms: mono-rhamnolipid and di-rhamnolipid with the di-rhamnolipid being the most abundant form.^17,21^ These are synthesized by the RhlB and RhlC proteins, respectively, from precursor 3-(3-hydroxyalkanoyloxy)alkanoic acids (HAAs), which are synthesized by RhlA.^17,22–25^ HAAs and RLs have variable lipid chain lengths, with over 30 distinct RL congeners having been identified in *P. aeruginosa*.^26^ RLs are amphipathic due to their hydrophobic lipid chain (3-hydroxyalkanoic acid) and hydrophilic sugar moiety (L-rhamnose).^17,27^ The amphipathic property enables RLs to function as surfactants, which are molecules that decrease the surface tension at interfaces. RLs and HAAs function additionally as wetting agents, which facilitate the spreading of liquid droplets on surfaces and have the effect of decreasing the contact angle that *P. aeruginosa* populations make with surface.^17,28,29^ RLs have been implicated in diverse roles due to their amphipathic properties.^26,30^ For example, RLs improve the ability of *P. aeruginosa* to uptake otherwise poorly soluble molecules such as hydrocarbons, which can be used as nutrient sources.^26,31–33^ RLs increase the solubility of the *P. aeruginosa* QS molecule 2-heptyl-3-hydroxy-4-quinolone (PQS),^34^ which could increase the diffusion distance of this cell-to-cell communication molecule. An important factor in pathogenesis, RLs are found in relatively high abundance in infection environments such as the lung, where they promote biofilm formation and inhibit host immunity.^26,35–37^

**FIG. 1.**
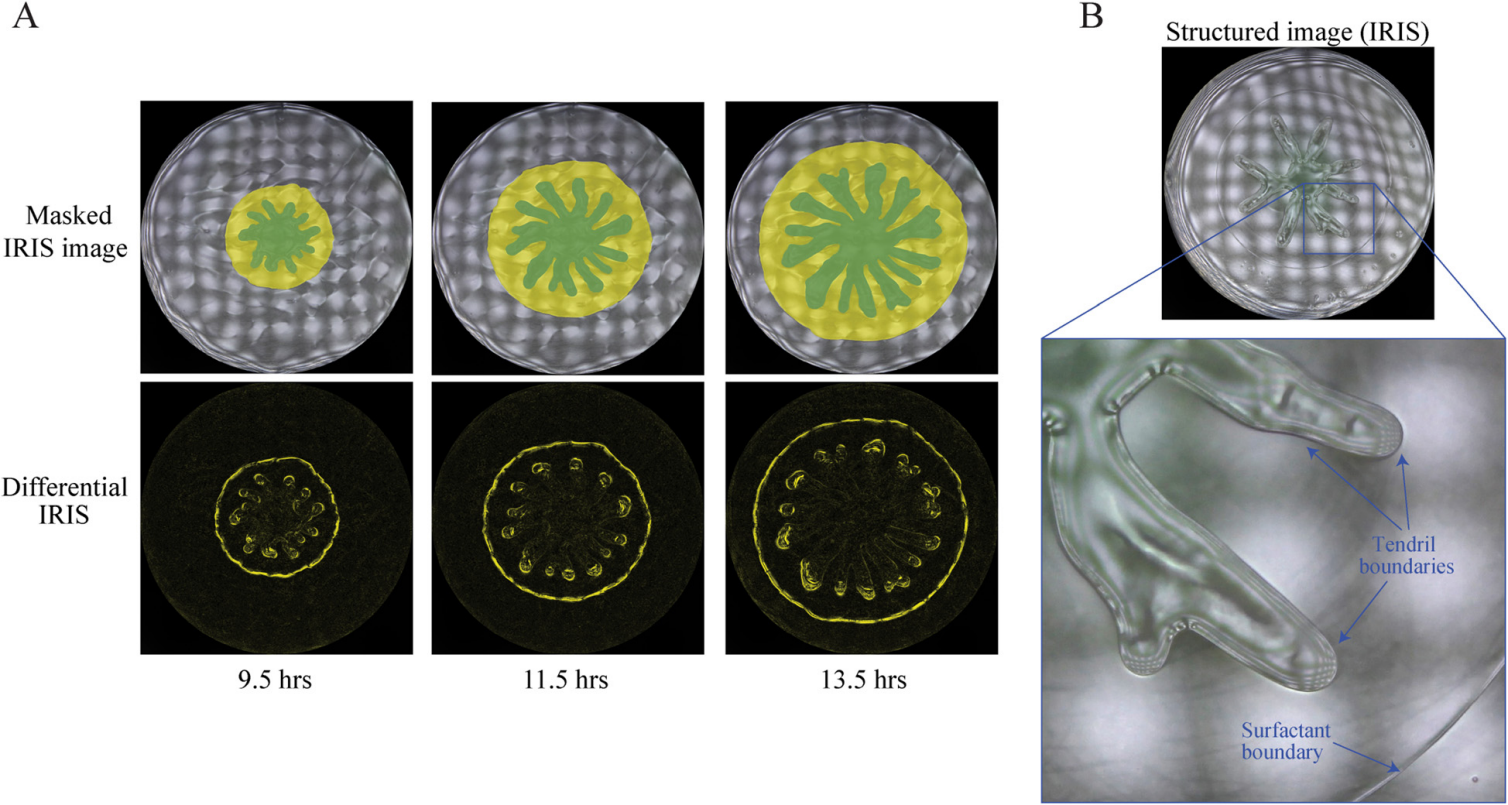
Expansion of a liquid-like zone precedes the outward growth of tendrils. (a) Images of swarms captured by imaging of reflected illuminated structures (IRIS) on 10 cm Petri dishes at 9.5, 11.5, and 13.5 hrs of growth. Masked IRIS images indicate tendrils consisting of bacteria (dark green) and the surfactant zone (yellow). Differential images indicate boundary movement (yellow) during 5-minute intervals. (b) IRIS image of a swarm and magnification indicating the surfactant and tendril boundaries. Reproduced with permission from Kasallis *et al.*, Curr. Opin. Solid State Mater. Sci. **27**(3), 101080 2023. Copyright 2023 Authors, licensed under a Creative Commons Attribution (CC BY).^43^

*P. aeruginosa* mutant strains that are defective in the production of RLs are deficient in swarm expansion and do not form tendrils.^38^ In contrast, RLs and HAAs produced by wildtype strains contribute to the formation a liquid-like zone that expands outward from the swarm and precedes the tendril growth^38–43^ (Fig. 1). The surfactant property of RLs reduces surface tension and friction between the bacterial population and the surface, facilitating the outward expansion of the population.^5,44^ However, the presence of a surfactant or wetting agent alone is not sufficient to explain how a swarm expands or how tendrils form. For example, in the case of *E. coli*, surfactant production is not required for swarming.^45,46^ While reduced surface tension and reduced friction appear to be important for swarming, a fundamental question that remains outstanding is what drives the swarm to expand outward in the first place? A potential explanation is that bacterial growth causes swarms to expand or that the activity of flagella drives the swarm expansion simply by propelling the bacteria forward. However, multiple studies reviewed in the Role of Fluid Mechanics in Swarm Expansion and Tendril Formation section suggest that growth and flagellar motility alone cannot account for the outward expansion of a swarm. They describe a more complex scenario that involves the movement of fluids from the agar substrate into the swarm itself.

### Regulation of RL and PQS production

The production of HAAs and RLs is regulated by QS, which is a bacterial cell-to-cell communication system. QS enables groups of bacteria to perform collective tasks including the production of biofilm components and virulence factors.^5,47–49^ QS ensures that HAAs and RLs are produced only when *P. aeruginosa* reaches high cell density.^50^ Specifically, QS is dependent on the release and population-wide detection of QS molecules known as autoinducers (AIs). At high cell density, AIs accumulate, and upon reaching a critical threshold, activate specific bacterial QS receptors, which in turn switch on group behaviors.

*P. aeruginosa* has multiple intertwined QS systems. The LasI synthase is considered to be at the top of the QS hierarchy. It produces the AI 3-oxo-C12-homoserine lactone, which activates its receptor LasR.^51–53^ LasR, in turn, drives expression of a number of genes, including the *rhlI *and* rhlR* genes.^54^ The AI N-butanoyl-L-homoserine lactone (C4-HSL) is synthesized by RhlI, the expression of which is additionally controlled by several small RNAs, including RsmA, RsmY, and RhlS.^55–57^ LasR and RhlR are the key QS regulators in this network.^52,58,59^ These regulators are activated when bacteria surpass a cell density threshold.^24,51–53,58^

RhlR is activated by C4-HSL. C4-HSL-bound RhlR activates transcription of *rhlAB* and *rhlC*,^24,58,60,61^ the products of which synthesize HAAs and RLs.^17,22,24^ The activity of RhlR is also enhanced by direct interactions with PqsE.^62–65^ Additional regulation of RhlR has been proposed to occur through the 2-(2-hydroxyphenyl)-thiazole-4-carbaldehyde (IQS) QS system.^66,67^ Together, this complex QS network ensures that RLs and HAAs are produced when *P. aeruginosa* reach high cell density.

LasR and RhlR together balance the production of the AI PQS.^68,69^ PQS has a role in swarming, but the mechanistic details of its involvement are unclear. Mutants that are defective in PQS production are defective in swarm expansion and do not form tendrils.^70^ PQS itself can also alter swarm patterns since tendrils are directly repelled by the molecule.^71^ PQS is bound by the transcriptional regulator PqsR, which regulates over 10% of *Pseudomonas* genes.^49,54,62,67,72^ Moreover, PQS can bind directly to hundreds of protein partners within the cell.^73^ In addition to its function as an AI, PQS is a cytotoxic agent, mediates iron acquisition and outer membrane vesicle biogenesis, and modulates host immune responses.^74^ The complex regulation of PQS by the Las and Rhl systems has posed a challenge for identifying environmental signals that regulate PQS production. Recent reports show that PQS production is increased by stress from antibiotics or bacteriophage infection, but the regulatory mechanism that links stress with PQS production is unclear.^71,75–77^

### Flagella—Tiny propellors

In addition to surfactants, a general requirement for swarming motility is the activity of the flagellum, which is a hairlike structure that rotates to produce motion.^78,79^ In many bacterial species, multiple flagella are produced during swarming.^45^ Typically a *P. aeruginosa* bacterium possesses a single polar flagellum that is critical for swarming, although some reports show that two flagella can be produced under specific conditions.^5,80^ *P. aeruginosa* variants that produce additional flagella give rise to “hyperswarmer” phenotypes in which an increased swarm expansion rate is observed,^81,82^ which underscores the important role of flagella in swarming.

The rate of flagellar reversals, in which a flagellum rotates in one direction then reverses in the other direction, is correlated with swarming such that a higher rate of flagellar reversals gives rise to hyperswarming.^83–85^ The number of flagellar reversals is controlled in *P. aeruginosa* by c-di-GMP, an intracellular signaling nucleotide that regulates the switch between swarming to biofilm lifestyles.^83,84,86,87^ Swarming and biofilm lifestyles are complementary, the former of which is an active (motile) lifestyle associated with low levels of c-di-GMP and latter of which are sessile (stationary) and have high levels of c-di-GMP.^86^ Swarming and biofilm formation can be viewed as opposite sides of the same coin that encompasses collective bacterial behaviors,^15,85,88,89^ which enable bacteria to build or move within structured environments. The manner in which flagellar reversals impact swarming is unclear. While flagellar activity is clearly important for swarming, a fundamental question that remains outstanding is how does flagellar activity contribute to the expansion of a swarm and tendril/dendritic formation? Are they simply tiny propellors? The swarm-enhancing effect of flagellar reversals suggest otherwise, which is discussed in the Role of Flagella in Swarm Expansion section.

## TENDRIL FORMATION AND SWARM EXPANSION

**FIG. 2.**
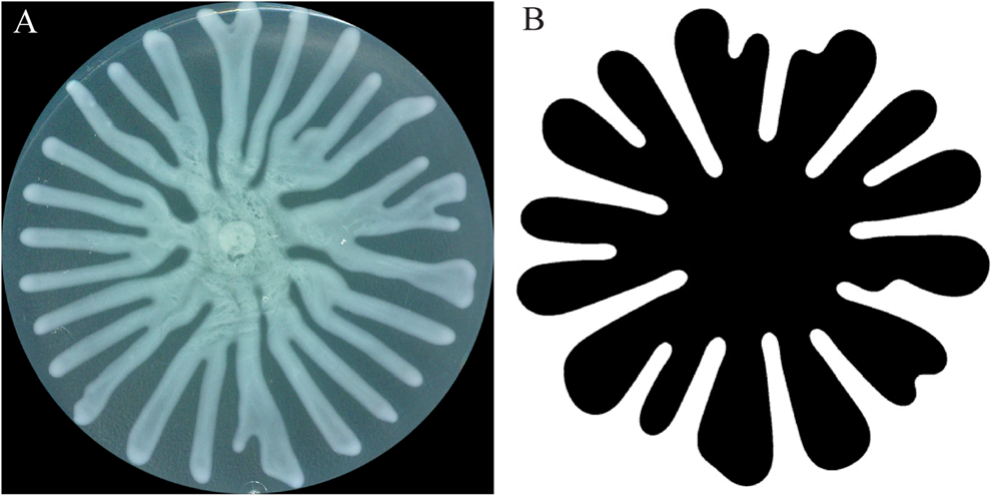
Tendril formation in *P. aeruginosa* and viscous fingering in an abiotic fluid. (a) A *P. aeruginosa* swarm develops from the inoculum at the center of a plate and forms tendrils on a 0.5% agar plate with M8 minimal media. Swarm imaged on a 10 cm Petri dish after 18 h of growth at 37 °C. (b) Abiotic fluids in a Hele–Shaw device form viscous fingering that resembles *P. aeruginosa* tendrils. (b) reproduced with permission from Ristroph *et al.*, Phys. Rev. E **74**(1), 015201(R) (2006).^94^ Copyright 2006 American Physical Society.

One of the hallmark features of *P. aeruginosa* swarms is the emergence of tendrils. Typically, multiple tendrils emerge and grow in length radially from the center of the inoculation [Fig. 2(a)]. There is remarkable diversity in *P. aeruginosa* tendril patterns, including variation in tendril morphology, number, length, thickness, and branching frequencies. This diversity has been attributed to a number of factors including media composition, surface hardness, moisture, growth conditions, and genetic backgrounds.^45,90–94^ How swarming patterns are altered by these factors and the mechanisms that govern the formation of tendrils remain a topic of debate.

We differentiate between swarm expansion and tendril formation to refer to two separate aspects of swarming. Specifically, we refer to swarm expansion as the property of the swarm to grow in size, regardless of whether tendrils are formed. On a semi-solid surface, this is observed as an increase in the surface area of the swarm. We refer to tendril formation as the development of dendritic patterns that can emerge in a swarm. Many studies do not distinguish between these different aspects, and in some cases, *P. aeruginosa* has been considered defective in swarming if tendrils are not present even if the colony has expanded and spread across a surface. In this section, we discuss mechanisms that contribute to swarm expansion and tendril formation, considering each as a distinct process.

### Role of chemotaxis—Movement of tendrils toward nutrients through sensing

The movement of tendrils toward areas of greater nutrient availability has raised the possibility that swarm expansion and tendril formation are a chemotactic response that promotes the search for nutrients. Indeed, iron and phosphate limitation and the presence of ethanol promote swarming in *P. aeruginosa*^14,96–98^ and the chemotaxis system is required for swarming in *E. coli.*^13^ In addition, swarming in *P. aeruginosa* is strongly dependent on the amino acids that are supplied as the nitrogen source.^5^ Computational simulations of nutrient gradients using reaction-diffusion equations and chemotaxis can reproduce many of the tendril features observed in experiments.^99–101^ Extensive nutrient depletion is required to create these gradients, which has drawn into question whether such models are representative of experimental conditions.^101^ Additional experiments in *E. coli* have demonstrated that while the chemotaxis system is needed, the chemosensor itself is not required for swarming.^13,102,103^ This supports the critical role of flagella function in swarming and suggests that direct nutrient sensing does not drive the outward expansion of tendrils. Rather, the chemosensory system is reprogrammed during swarming to increase the distance of movements before changing direction, resulting in trajectories known as Lévy walks, which have the potential to maximize search of space.^104^ In this model, the chemotaxis system operates in a “locked on” mode that is insensitive to chemical gradients. These data suggest that while specific nutrients are required for swarming, nutrient sensing is not the primary driver of tendril movements.

### Movement of tendrils based on sensing of QS-associated products

*P. aeruginosa* tendrils repel neighboring tendrils. This property raises the question of how *P. aeruginosa* regulates the direction, movement, and boundary of its tendrils. RLs have been proposed to coordinate tendril formation due to their concentration gradients.^38^ In this model, the lowest concentrations of RLs enable the linear movement of tendrils and higher concentrations result in movement inhibition. This model explains why tendrils move toward areas devoid of RLs and are repelled from neighboring tendrils. Further support of this model is the involvement of SadB in sensing RLs.^38^ Tendrils of *sadB* mutants do not change direction in response to approaching tendrils and move through RL zones. In such a model, tendrils move forward linearly due to the sensation of lower concentrations of RLs at the tendril tips and are inhibited from lateral movements at the side due to sensation of high concentrations of RLs from neighboring tendrils. While SadB is involved in the sensing of RLs, the details of the sensory mechanism are unclear.

An additional model posits that di-RLs and HAAs have opposing roles: di-RLs attract swarming cells and promote tendril formation whereas HAAs are strong repellants and inhibit tendril formation.^105,106^ The tendril pattern is, thus, a result of competition between opposing forces. A report demonstrated that RLs at concentrations above 2.5 mM attract swarm tendrils, but below this concentration repel tendrils, suggesting a concentration-dependent effect for RLs.^71^

Tendrils are repelled by the AI PQS and to a lesser degree by the PQS precursor molecule 2-heptyl-4-quinolone (HHQ).^71^ These molecules are over-produced in *P. aeruginosa* colonies that are treated with antibiotics or that are infected by bacteriophage.^71,76^ This has raised the possibility that *P. aeruginosa* tendrils can physically avoid areas where there is a stressor present. PQS and HHQ bind the QS regulator PqsR,^73,107^ which represents a potential sensor for tendril movement control. Consistent with the stressor hypothesis, a recent report demonstrated that *P. aeruginosa* swarms are repelled by QS controlled phenol soluble modulin fibers that are produced by *S. aureus.*^42^ The putative pilus chemoreceptor PilJ in *P. aeruginosa* detects the presence of *S. aureus* and could be involved in sensing phenol soluble modulins.^108^ Together, these reports demonstrate that tendrils are repelled by a number of QS-associated molecules and suggest that distinct sensors could detect the diverse molecules. It is possible that a signaling pathway integrates input from multiple sensors or that the sensory information is transduced through individual networks. Regardless of how the sensory information is processed, a significant mystery remains: it is unknown how *P. aeruginosa* could alter tendril movement in response such that they are steered around an area abundant in a particular molecule.

Potential insight into how tendril movement is controlled comes from an observation that tendrils are repelled by a number of distinct molecules including linoleic acid, oleic acid, and the abiotic lubricant polydimethylsiloxane.^42^ It is unclear how a sensory system could detect such a broad range of molecules and modulate swarm patterns in response. The ability of *P. aeruginosa* swarms to respond to both biotic and abiotic factors raises the possibility that tendril formation is guided by an additional or alternative mechanism that does not require direct detection by a sensory system, which is discussed in the following sections that describe the role of fluid mechanics.

## ROLE OF FLUID MECHANICS IN SWARM EXPANSION AND TENDRIL FORMATION

*P. aeruginosa* in swarms are densely packed within a viscous liquid medium that includes water, HAAs, and RLs. Fluid mechanics governs the dynamics of liquid media, but to what extent do fluid mechanics-based mechanisms contribute to swarm expansion and tendril formation? To answer this question, one can investigate the dynamics of abiotic fluids. Tendril formation is observed in abiotic fluids that spread on solid surfaces. For example, abiotic tendrils are formed by a process referred to as viscous fingering [Fig. 2(b)] and Saffman–Taylor instabilities.^19,109–112^ Here, a fluid that is driven by pressure displaces another fluid of higher viscosity, forming tendrils or fingers in the process. This phenomenon can be observed in a Hele–Shaw device, in which a fluid is confined between two plates. A second fluid of lower viscosity is supplied to the center of the device and slightly pressurized such that it flows radially outward between the plates and forms tendrils in the process [Fig. 2(b)]. The shape and dynamics of tendrils formed in Hele–Shaw devices have remarkable resemblance to *P. aeruginosa* tendrils observed in swarms [Fig. 2(a)]. Critical tendril properties, including finger number and length, and number of branch points, can be altered in abiotic fluids to resemble patterns observed in swarm patterns produced by diverse bacteria.^95,113,114^ The formation of swarm-like tendrils by abiotic fluids raises the possibility that fluid mechanics could have an important role in the formation of swarm patterns. Two fluidic processes specifically have been discussed: Marangoni-driven flow and pressure-driven flow. Both models consider bacterial swarms to be thin liquid films and recapitulate swarm expansion and tendril formation. However, the mechanisms responsible for these effects are markedly distinct. A notable feature of both models is that they do not rely on biological sensing mechanisms such as RL sensing or chemotaxis.

### Contribution of Marangoni flow

**FIG. 3.**
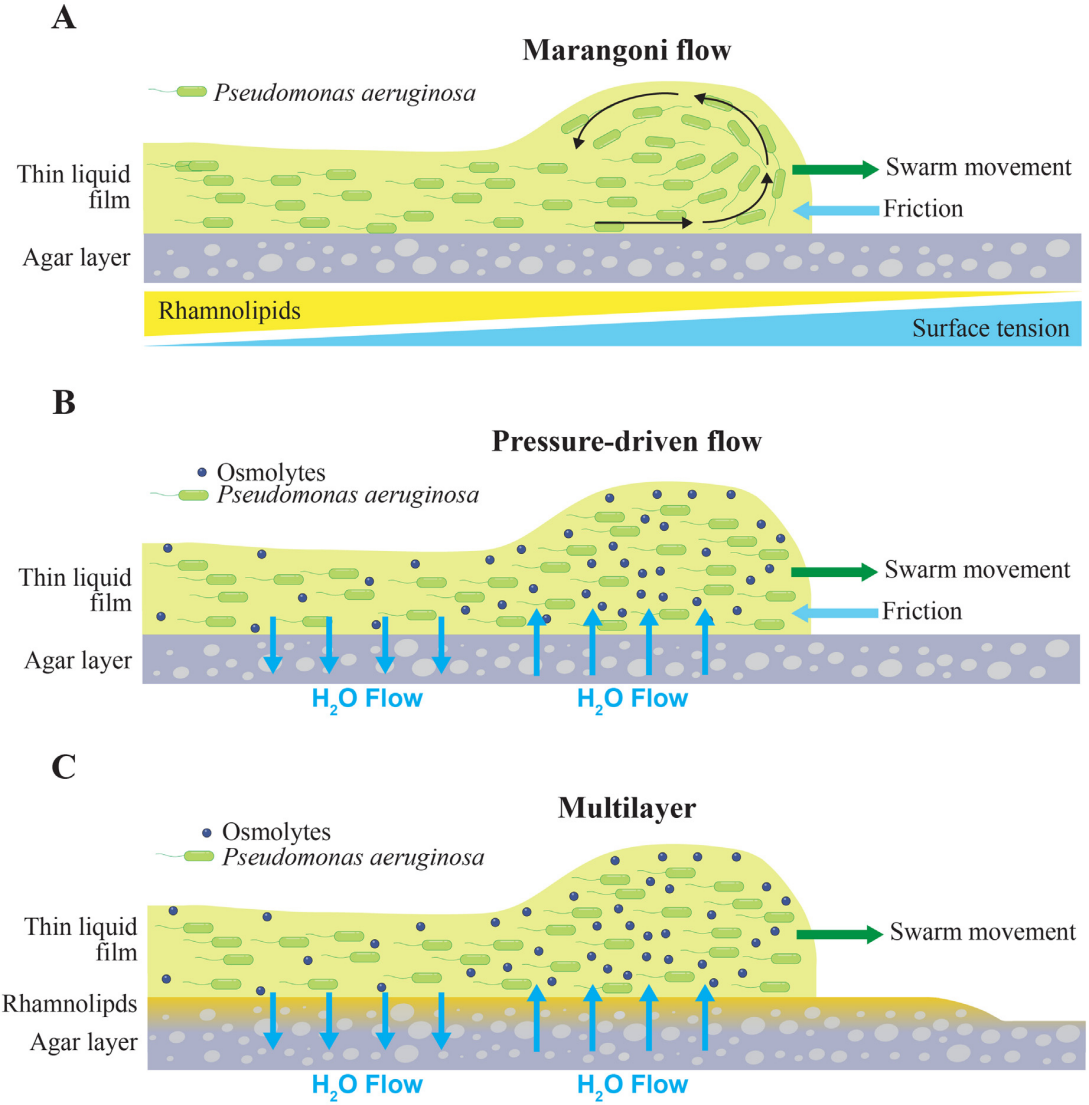
Schematics of Marangoni flow, pressure-driven flow, and multilayer models of swarming. (a) A gradient of rhamnolipid concentration creates a surface tension gradient, which induces Marangoni flow. This flow runs cyclically within the bacterial population, which is treated as a thin liquid film, and drives the expansion of the swarm. Schematic based on Ref. 118. (b) The production of osmolytes by bacteria induces a flow of liquid from the agar layer into the layer containing bacteria near the swarm edge. The influx of liquid creates a pressure that causes the bacterial population to expand. Some liquid flows back into the agar, creating a circulating flow. Adapted from figures in Refs. 119 and 120. (c) Incorporation of rhamnolipids into the pressure-driven flow model. The bacterial population expands on the surface of a layer that contains rhamnolipids.

Marangoni flow arises when a gradient of surface tension is present in a liquid and is due to a fluidic effect in which liquid flows in a direction from lower to higher surface tension. A notable example of Marangoni flow is the “tears of wine” phenomenon in which wine droplets move along a wine glass surface upward, where the surface tension is higher, and drop back into the body of wine. In swarms, bacteria are embedded in a viscous liquid medium that is bounded by surface tension at an air-liquid interface. Surface tension arises from the cohesive forces in the liquid medium and opposes the expansion of the bacteria-containing liquid medium [Fig. 3(a)]. The production of surfactants by bacteria could create a surface tension gradient within the swarm.^115^ The gene expression of RL-producing enzymes in *P. aeruginosa* decreases from the center of the swarm toward the swarm front,^116,117^ suggesting that a surface tension gradient is present. In particular, the lowest surface tension would be in the center of a swarm and the highest surface tension at the swarm front.^116^ This condition would produce a Marangoni flow in which the bacteria-containing liquid flows from the center of the swarm toward the front edge, which would propel the outward expansion of the swarm [Fig. 3(a)]. Consistent with this model, Marangoni flows can run cyclically within the liquid medium of a colony and have a significant impact on colony morphology, causing bacteria to move to the edge or to the center of a colony.^118^ In addition, tendrils can develop due to the non-linearity of Marangoni flows and additional parameters that impact their dynamics (i.e., concentration gradients, dynamic surface tension, and viscosity). Small perturbations at the edge of the swarm front can be amplified, resulting in relatively large tendrils.

Through computational simulations, Du *et al.* showed that tendrils could form from Marangoni forces that arise from the production of RLs.^121,122^ The authors found that densely packed *P. aeruginosa* increased the viscosity of the liquid film, thereby reducing the film's spreading speed. RLs created a surface tension gradient that pushed the liquid film and *P. aeruginosa* outward. Small perturbations at the swarm boundary were amplified by Marangoni forces and resulted in tendril formation. Trinschek *et al.* demonstrated the emergence of swarm tendrils by implementing a model that incorporated the effects of surface wettability and Marangoni flows due to surfactants.^123^ This model incorporates a passive thin liquid film that is driven by a growth law and production of surfactant to produce a diverse range of growth phenotypes, including swarms without distinct tendrils and those with pronounced tendrils. Modification of this model to incorporate chemosensing and motility showed that swarm tendrils could detect and be repelled by antibiotics.^124^ While these reports have demonstrated that swarm tendrils and the advancement of the swarm front can be explained by Marangoni flows, it is unclear whether this is the primary mechanism that drives swarm expansion or tendril formation.

### Contribution of pressure-driven flow

An additional fluid mechanics-based mechanism that may contribute to swarming relies on a process that is related to the formation of tendril-like fingers in abiotic fluids in the Hele–Shaw device [Fig. 2(b)]. In this device, the expansion of the fluid and formation of tendrils are driven by pressure that is supplied from an external source. In fluid mechanics, the movement of fluid by pressure (pressure-driven flow) is governed by a relation known as Darcy's law. Giverso *et al.* investigated the impact of pressure-driven flow on tendril formation by bacterial swarms.^18,125,126^ In their model, the bacterial swarm is treated as a thin liquid film that is surrounded by ‘lubricant’ fluid present on the Petri dish. The swarm expansion is driven by an internal pressure, which is described by Darcy's law. A critical parameter of the model is how internal pressure arises in the swarm. The authors considered two models that are described by mass balance equations: volumetric expansion and chemotactic growth.

In the volumetric expansion model, nutrient supply facilitates bacterial growth, which creates a pressure in a swarm that drives the population expansion outwards. The extent of tendril formation is governed by pressure created by chemomechanical interactions that arise due to energy from nutrients, surface tension, and friction, and the size of the lubricant fluid zone. While the model was not developed specifically for *P. aeruginosa*, it could describe swarming of this species. In particular, *P. aeruginosa* swarms contain two distinct zones that contain liquid–air interface boundaries: one zone formed by the bacterial cells and another zone containing surfactant [Fig. 1(b) and Refs. 41 and 43). In the context of the volumetric expansion model of Giverso *et al.*, the surfactant zone produced by *P. aeruginosa* could be considered the lubricant fluid. In this interpretation, swarm expansion in *P. aeruginosa* would be driven by pressure created by growth. Tendril formation would arise from chemomechanical interactions between *P. aeruginosa* and the growth medium surface and would be affected by the surfactant zone size.

In the chemotactic growth model, pressure in a swarm is created by the chemotactic motility of bacteria toward an attractant. Simulations of the volumetric and chemotactic models yield tendrils that are qualitatively similar to experimental observations but have quantitative differences such as the translation of center of mass of swarms. In light of experimental evidence suggesting that chemosensing is dispensable for swarming,^13,104^ the volumetric expansion model may be more relevant for tendril formation in *P. aeruginosa.*

### Volumetric expansion driven by fluid influx

At the heart of the volumetric expansion model is the premise that nutrients and other precursor materials must be transported into the swarm in order for it to expand. As RLs and HAAs are solid themselves, the bulk of the surfactant layer consists of fluid. The expansion of the surfactant and bacterial layers in *P. aeruginosa*, thus, requires transport. From where and how does this transport occur? In the case of *E. coli*, Wu *et al.* examined the role of fluid influx from the agar layer into the swarm as a driver of swarm expansion.^119^ The authors tracked the fluid flow in swarms using microbubbles. They identified the presence of fluid reservoirs near the leading edge of *E. coli* swarms and proposed a model in which water is drawn up from the agar layer into the bacterial layer at the leading edge and pumped outward by flagella, resulting in swarm expansion. The authors suggest that the increase in cell density and metabolism by-products function as osmolytes that drive the water flux from the agar layer to the bacterial layer. Ping *et al.* measured spatial changes in osmolarity in *E. coli* swarms using liposomes and confirmed a significant increase in osmolarity at the leading edge of the swarm.^127^ The authors suggest that the osmolytes are relatively high in molecular weight and could be lipopolysaccharides. Srinivasan *et al.* performed simulations based on the experimental work of Wu *et al.* and Ping *et al.*, referring to the model as a “steady state” swarming model, which was based on the assumption that bacteria produce osmolytes.^120^ Here, Darcy's law appears again but describes the flow of fluid between the agar and bacterial layers. The presence of osmolytes in the bacterial layer near the swarm edge induces fluid to flow from the agar layer into the bacterial layer by capillary force and viscous stresses [Fig. 3(b)]. In locations distant from the swarm edge, some of the fluid is returned into the agar layer. The recirculating flow between the agar and bacterial layers results in overall swarm expansion [Fig. 3(b)].

The entry of water into the bacterial layer described by these models causes volumetric expansion of the swarm, similar to the volumetric expansion model proposed by Giverso *et al.* The notable difference is that Wu *et al.*, Ping *et al.*, and Srinivasan *et al.* attributed the swarm expansion to the flow of fluid from the agar layer into the bacterial layer, whereas Giverso *et al.* attributed the swarm expansion to bacterial growth. It is also relevant to note that these studies focused on *E. coli*, which does not produce surfactant. In addition, with the exception of Giverso *et al.*, these studies did not provide a mechanism for how fluid influx contributes to tendril formation.

What causes the influx of fluid into the bacterial layer? Yang *et al.* investigated the role of fluid flux between the agar and bacterial layers in swarm expansion in *P. aeruginosa.*^128^ The study posited that osmolytes such as lipopolysaccharides that are produced by *P. aeruginosa* create osmotic pressure that draws fluid from the agar layer into the bacterial layer, which is governed by Darcy's law. Their experiments showed that increasing osmolarity in the agar layer decreased swarming motility, which can be understood by a reduced osmolyte differential between the bacterial and agar layers. The reduced osmolyte differential results in decreased flow to the bacterial layer and concomitantly, decreased expansion of the swarm. These findings support a significant role for fluid influx from the agar layer into the swarm layer and a role for pressure-driven flow in *P. aeruginosa* swarming.

### Importance of fluid transport

A fundamental assumption in the fluid influx models is that flagella-driven motility alone cannot cause the volumetric expansion of swarms. To understand why motility alone cannot expand a swarm, consider that the bacteria are enveloped in liquid, the boundary of which is a liquid-air interface [Fig. 1(b)]. Individual bacterial cells cannot cross over from the liquid onto other areas of the growth medium surface, because they are bound by relatively high surface tension. Bacterial cells are thus constrained to move within the bounds of the liquid, such that swimming within the liquid does not increase its volume. An example that illustrates this scenario is the growth of bacteria in a flask. Bacteria can swim within the liquid medium contained in a flask but cannot leave the liquid medium. Swimming within the liquid medium, no matter how active, will not expand the volume of the medium contained within the flask. In order to achieve volumetric expansion, additional materials would need to be added to the medium in the flask. In the case of swarming, the volumetric expansion of the swarm requires additional materials. In the models presented above, this addition is due to the transport of fluid and nutrients from the agar layer into the bacterial layer.

### Clarification of Darcy's law

It is noted that Darcy's law, which describes flow that is induced by pressure, is used to describe multiple processes related to swarming. Giverso *et al.* described internal pressure that drives the expansion of the swarm and can result in the formation of tendrils.^126^ Srinivasan *et al.*, based on the analyses of Wu *et al.* and Ping *et al.*, arrived at a Darcy law-like model to describe the influx of fluid into the *E. coli* swarm from the agar layer, driven by osmotic pressure.^119,120,127^ Similarly, Yang *et al.* described the influx of fluid into the *P. aeruginosa* bacterial layer using Darcy's law.^128^ Swarming in *P. aeruginosa* could involve the coupling of two processes that are described by Darcy's law: fluid transfer between the agar layer and bacterial population and volumetric expansion due to the influx of water. Such a mechanism that couples these two processes could in principle give rise to tendril formation in *P. aeruginosa*.

### Relative contributions of Marangoni and pressure-driven flows

To what extent can swarming be attributed to pressure-driven flow or to Marangoni flow? Yang *et al.* added the surfactant Triton X-100 to the swarm agar to decrease the surface tension gradient.^128^ The Triton X-100 was expected to rise above the agar surface and over the swarm fluid due to the strong preference that surfactants have for liquid-air interfaces.^129^ A decrease in this gradient was expected to decrease Marangoni flows, and thus decrease swarm growth and tendril formation. However, the opposite was observed, with the addition of the surfactant enhancing swarm growth and tendril formation. The authors rationalized that the result implies that swarming is not driven predominantly by Marangoni flow but rather by a mechanism that is consistent with pressure-driven flow. In this model, surface tension contributes to a frictional force that opposes the growth of the swarm. Consistent with this, Ke *et al.* found that surface tension restricted swarm growth in *B. subtilis.*^130^ Decreasing surface tension relieved the opposing force and facilitated enhanced swarm growth. Importantly, the study of Yang *et al.* showed that the surface tension gradient is dispensable for swarming, which suggests that Marangoni flow is not the primary driver of swarm growth or tendril formation. The relative contribution between Marangoni flows and pressure-driven flows in *P. aeruginosa* swarms remains to be determined.

### Role of surfactant flow in swarm pattern development

We consider the role of RLs in the pressure-driven flow model. RLs and HAAs contribute to the formation of a liquid-like zone that is distinct from the zone containing the bacterial population^38–42^ (Fig. 1).^43^ Imaging of swarms using the IRIS technique, which is a noninvasive method of tracking surfactant and tendril expansion in swarms, shows that the liquid-like zone and bacterial population have distinct heights relative to the agar surface and appear as separate layers.^43^ Measurements using optical profilometry showed that the liquid-like zone has a greater height than the agar surface but is below the bacterial layer.^41^ Additional experiments using shadowgraphy suggest that RLs produced by *P. aeruginosa* imbibe the agar, causing it to swell,^41^ though the concentrations of RLs within the agar matrix compared to the agar surface are unclear. If RLs are incorporated into the pressure-driven model, we arrive at a multilayer model in which RLs are present at the agar surface and potentially within the agar matrix [Fig. 3(c)]. We refer to the liquid-like zone as a surfactant layer due to its dependence on surfactant production and its appearance as a distinct layer. While the surfactant layer is likely to contain RLs, the concentration of RLs relative to water, agar, and HAAs is unclear and requires characterization.

**FIG. 4.**
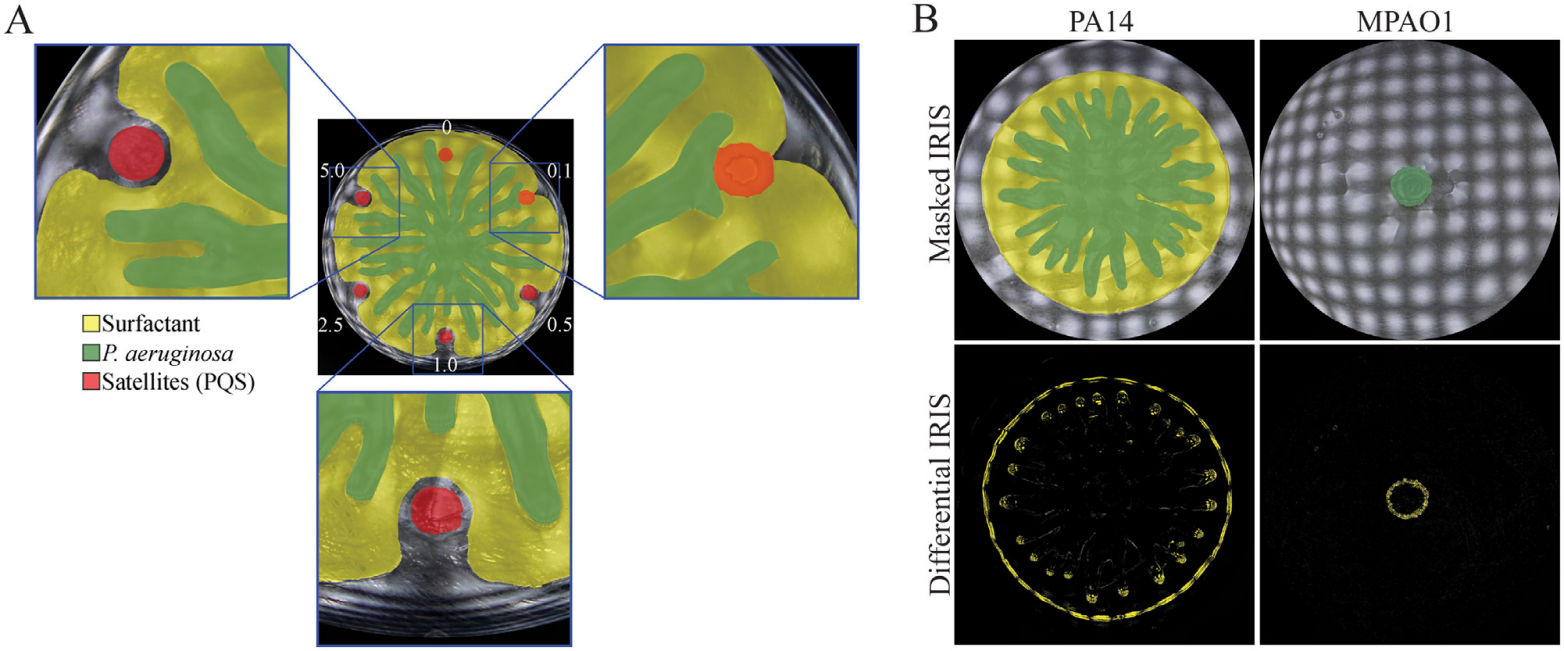
Diverse swarm patterns in *P. aeruginosa*. (a) PQS alters tendril movement by deflecting the surfactant layer of PA14. The concentration of PQS in millimolar is given. (b) IRIS images of PA14 and MPAO1 at 12 h following inoculation. Masked IRIS images indicate tendrils consisting of bacteria (dark green) and the surfactant layer (yellow). Differential images indicate boundary movement (yellow) during a 5-minute interval. Experiments were performed using 10 cm diameter Petri dishes and M8 swarm medium. (a) reproduced with permission from Kasallis *et al.*, Curr. Opin. Solid State Mater. Sci. **27**(3), 101080 2023. Copyright 2023 Authors, licensed under a Creative Commons Attribution (CC BY).^43^

The presence of bacteria and RLs in distinct layers raises the possibility of long-range effects. For example, RLs from one colony can alter the swarm patterns of a distant colony even if the bacteria in different colonies do not make physical contact with each other.^41^ A converse long range effect can also occur in which the alteration of RLs alters the movement of the bacterial population.^43^ For example, the surfactant layer can be deflected by a number of molecules including oleic acid, the abiotic lubricant PDMS, and the QS molecule PQS [Fig. 4(a)].^42^ The deflection of the surfactant layer by these molecules alters the movement of tendrils. This effect is due to the constraint that tendrils move only on the surfactant layer. If the surfactant layer is deflected, the tendrils are constrained to move only within the boundaries of the altered surfactant layer. These results suggest that tendril movement can be altered by a broad range of molecules without the use of a sensory system, but rather through changes in the surfactant flow. While the data show that surfactant flow is a major contributor to tendril formation, they do not rule out a role for a sensory-response system. It is possible that surfactant flow and sensing of QS-associated products work together to alter tendril movements.

### Swarming in PA14 and PAO1

Diverse swarm patterns have been observed among different *P. aeruginosa* strains. Understanding how different swarm patterns arise within a single species could provide insight into the role of the fluid mechanics-based mechanisms described above in tendril formation. In particular, the *P. aeruginosa* strains PA14 and PAO1 produce different swarm patterns. Most reports of PA14 show this strain producing swarm patterns that contain tendrils.^43,84,131,132^ In contrast, PAO1 produces a spectrum of swarm patterns, including those that are dendritic and fractal-like^93,133–136^ and patterns that are circular but do not contain prominent dendritic patterns.^94,131,132,134,137^ The diversity of swarm patterns could be attributed to the different growth conditions in studies, including the use of different growth media; the concentration of agar; and the moisture content of the swarm plates.^93^ For example, increasing the drying time of a swarm plate by 10 minutes can drastically alter swarm patterns.^93^ Direct comparisons of swarm patterns between the PA14 and PAO1 on the same medium^93,94,131,132,138^ are compounded by the use of multiple PAO1 lineages, including PAO1-UW, MPAO1, and PAO1 ATCC, which each have different genomic elements.^139^ These factors have created a challenge for identifying the processes that differentiate PA14 and PAO1 swarm patterns.

We documented the role of the surfactant flow in PA14 and the PAO1 strain MPAO1 using IRIS imaging. Growth on M8 minimal medium produced swarm tendrils in PA14 and a disk-like pattern with ruffled edges and no swarm expansion in MPAO1 [Fig. 4(b)], results which are consistent with previous observations.^131,138^ The masked and differential IRIS images indicate the presence of two expanding zones in PA14: a zone containing bacteria in the shape of tendrils, and a zone of fluid consistent with a surfactant layer [Fig. 4(b)]. In contrast, only the bacterial layer is present in MPAO1 and it is significantly smaller than that of PA14. The absence of a surfactant layer in MPAO1 is consistent with decreased production of RLs on surfaces by this strain.^138^ The presence of the RL layer facilitates tendril formation and swarm expansion in PA14 whereas its absence in MPAO1 reduces these features. Consistent with this interpretation, prominent tendrils are not observed in RL-deficient mutants of PA14 because they produce little or no surfactant.^38,42^

### Involvement of sliding in *P. aeruginosa* swarming motility

**FIG. 5.**
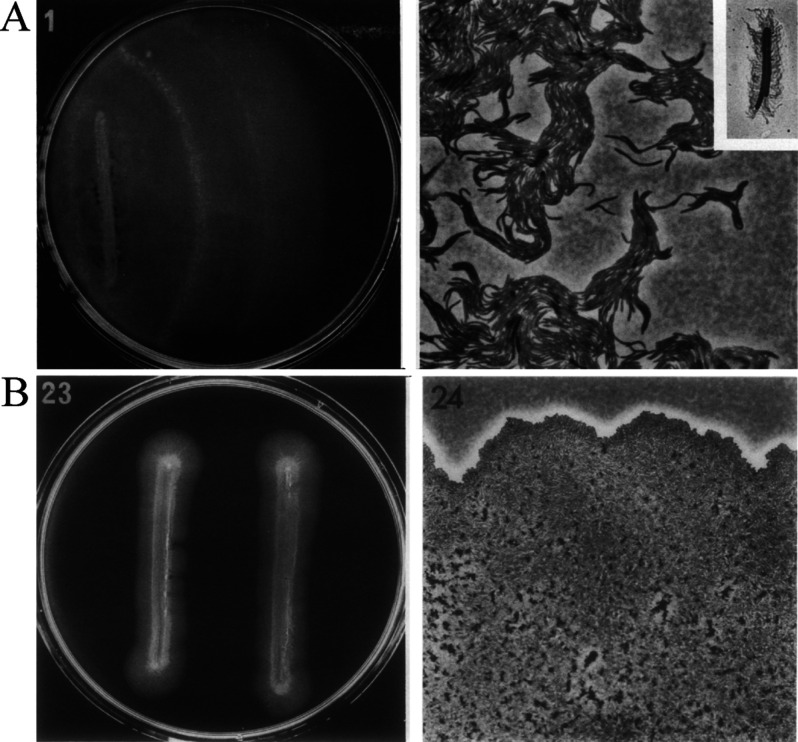
Micromorphologies of swarming and sliding bacteria. (a) *Proteus mirabilis* swarms form bundles or cell aggregates at the swarm edge using flagella. (b) *Acinetobacter calcoaceticus* exhibits sliding motility, in which cells move together along the surface as a single uniform layer of cells. Reproduced with permission from Henrichsen, Bacteriol. Rev. **36**(4), 478–503 (1972). Copyright 1972 American Society for Microbiology.^140^

Many assessments of *P. aeruginosa* swarming have been based on macromorphological features such as tendrils observed on Petri dishes without the use of optical magnification. Such assessments of swarming differ from earlier definitions, which were based instead on micromorphological features that require single-cell resolution microscopy of bacteria at the swarm edges.^44,140^ In particular, Henrichsen precisely defined in 1972 a number of bacterial translocation modes, including swimming, swarming, and sliding.^140^ Swimming and swarming were defined as surface translocation that are driven by flagella. Swarming differs from swimming because individual cells aggregate in bundles during swarming [Fig. 5(a)], whereas swimming cells do not have an obvious micromorphological organization. Sliding was defined as surface translocation due to expansion of the culture by growth, in combination with reduced friction between the cell and semi-solid surface. Sliding differs from both swarming and swimming because it does not require the activity of flagella. The micromorphological feature of sliding is that cells move together as a single uniform sheet of cells [Fig. 5(b)].

Swimming is readily observed in *P. aeruginosa* due to its robust flagellar activity. However, the distinction between swarming and sliding motilities in *P. aeruginosa* are less clear. Murray *et al.* observed sliding motility in strains that are deficient in pili and flagella (*pilA fliC*).^134^ The colony expansion was further reduced in this background by introducing a *rhlA* mutation, which disrupts the production of RLs. The ability of *P. aeruginosa* to expand without flagella and with the assistance of RLs is consistent with Henrichsen's description of sliding motility in these mutants, although micromorphological features were not reported in the study.

The bulk of the data on swarming by wildtype *P. aeruginosa* is challenging to evaluate against Henrichsen's definition. An early report concluded that multiflagellated mutants of *P. aeruginosa* exhibited swarming, which was based on the increased diameter of colonies on Petri dishes rather than micromorphological features within swarms.^141^ Two other early reports of *P. aeruginosa* swarming by Köhler *et al.* and Rashid and Koernberg placed emphasis on macromorphological features and the dependence of the phenotypes on flagella.^5,133^ While the three studies showed single-cell resolution images of cells from the swarm edge, the effect of the resuspension techniques on micromorphological features is unclear. The strong dependence of swarm expansion and tendril formation on flagella are suggestive of a swarm phenotype. Köhler *et al.* additionally described the dependence of the phenotype on pili.^5^ However, the dendritic patterns have also been observed in strains of *P. aeruginosa* that lack pili.^128,136^

**FIG. 6.**
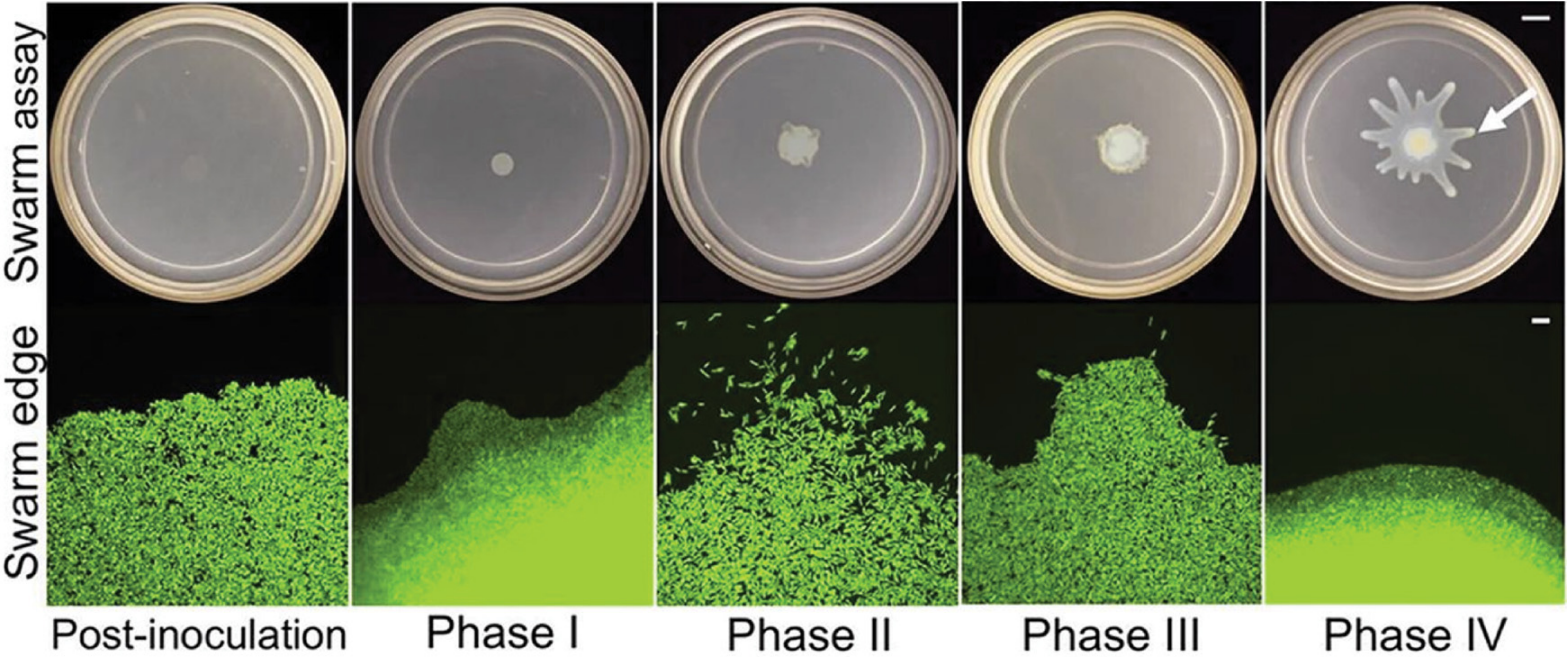
Macromorphological and micromorphological features of *P. aeruginosa* during swarm development. Micromorphological features of *P. aeruginosa* swarms are different at the colony/tendril edge during different points of swarm development. Phases I and IV show community expansion, resembling sliding motility. Phase II shows high bacterial motility, resembling flagella-dependent motility. The scale bar on swarm assay images is 10 mm and for swarm edge images is 10 *μ*m. Reproduced with permission from Madukoma *et al.*, J. Bacteriol. **201**(19), e00184 (2019). Copyright 2019 American Society for Microbiology.^142^

More recently, Madukokma *et al.* investigated micromorphologies and the dynamics of *P. aeruginosa* at the edge of expanding colonies in swarming conditions.^142^ The authors identified four phases of colony expansion that are marked by distinct morphologies and kinematics (Fig. 6). Phase I is marked by uniform community expansion and low single-cell velocities. Phase II has the highest single-cell velocities, and phase III has the highest community expansion. Phase IV, which marks tendril formation, is characterized by lowered community expansion and decreased single-cell velocities. The presence of multiple phases suggests that multiple motility mechanisms may be at work in *P. aeruginosa* swarms. In particular, community expansion during phases I and IV are reminiscent of sliding motility described by Henrichsen^140^ whereas the high motility during phase II is consistent with flagellar activity associated with swarming. A notable observation is the lack of organized aggregates, which are a defining feature of swarming.^140^ It does not appear that swarming by *P. aeruginosa* conforms to the precise description of swarming laid out by Henrichsen. It is possible that *P. aeruginosa* swarms reflect the convolution of multiple mechanisms associated with both swarming and sliding. The requirement for flagella suggests a swarming-like mechanism whereas the requirement for surfactant production suggests a sliding-like mechanism. The interplay between both swarming-like and sliding-like mechanisms may be responsible for the expansion and tendril patterns that emerge.

### Metrics of surface motility

Given that Henrichsen's definition of swarming is based on criteria at the single-cell level, it is not feasible to evaluate whether strains form swarms using macromorphological features alone. Despite this conflict, the appearance of tendrils has been frequently used as a criterion to evaluate whether a strain has formed a swarm, in part due to the simplicity of the assay on Petri dishes. The evaluation of whether a strain has formed a swarm is particularly challenging in situations in which strains form short tendrils. For example, some strains produce rough boundaries at the edge of growth that resemble tendrils in the early stages of formation,^94,131,132,134,137^ such as those present in MPAO1 [Fig. 4(b)]. In such cases, it may unclear whether the strains are exhibiting diminished swarming motility or lack swarming motility altogether. We propose the use of quantitative metrics to evaluate macromorphological features and more precisely classify observations of surface motility. Such metrics would provide a continuum of measurements of surface motility rather than binary labeling of an observation as a swarm or not a swarm.

For example, two metrics that could be helpful are the tendril-to-disk ratio and surfactant-to-disk ratio. These metrics rely on the observation that *P. aeruginosa* forms a colony in the shape of a circular disk at the center of the swarm regardless of tendril formation. The outer edge of the center disk thus provides a reliable reference point. The tendril-to-disk ratio can be computed by dividing the average tendril length, as measured between the center disk and the tip of the tendril, by the radius of the center disk. This ratio provides a unitless measure of swarm shape and patterning. Strains that produce long tendrils such as PA14 would have tendril-to-disk ratios greater than one. Those that produce flare-like edges such as some PAO1 strains would have ratios that are less than one. The surfactant-to-disk ratio is an additional metric that can be computed by dividing the average length of the surfactant boundary from the center disk by the radius of the center disk. This ratio provides a unitless assessment of surfactant production. Strains that produce copious amounts of surfactant such as PA14 could have ratios that are greater than one. Strains that produce little surfactant on surfaces such as MPAO1 would have ratios that are much less than one. As the metrics are unitless, they could be used to assess surface motility patterns across different *P. aeruginosa* strains and a wide range of conditions. Moreover, such metrics could be applied to different bacteria to improve the classification of swarming across multiple species.

## ROLE OF FLAGELLA IN SWARM EXPANSION

Flagella have a critical role in promoting swarm expansion and tendril formation. For example, *P. aeruginosa* that are deficient in flagellar activity produce colonies that are significantly reduced in size, do not form prominent tendrils, and have rough edges that resemble early stage tendrils.^38^ The presence of sub-populations of *P. aeruginosa* that are deficient in flagellar activity can repress swarming in the entire population.^143^ On the other hand, hyperflagellated *P. aeruginosa* and other bacteria produce enlarged swarms.^81,141,144,145^ Despite the important role of flagella in swarms, there is a significant knowledge gap regarding how flagella promote swarming. Multiple effects of flagella have been considered. In *Proteus mirabilis* and *Bacillus subtilis*, the formation of side-by-side cell groups known as rafts facilitate swarming.^2,146^ These rafts are thought to arise through bundling of multiple flagella with neighboring cells. However, raft formation has not been observed in *P. aeruginosa*. Flagellar reversals could help cells escape confined environments or increase the outflow of cells across the edge of swarms.^147–151^ In addition, flagella bending decreases the viscosity of the local environment,^152^ which could increase the outward movement of the swarm. It is important to note that while these effects could increase the diameter of a swarm, they could not cause volumetric expansion. To achieve volumetric expansion, an influx of nutrients and liquid from the agar into the swarm is required.

How could the activity of flagella increase the influx of nutrients and liquid into the bacterial layer? Multiple models have been proposed in which the activity of flagella increases the concentration of osmolytes in the bacterial layer. In *S. enterica* serovar Typhimurium, it has been proposed that flagella sense and generate wetness.^102,103,153^ In this model, the flagellum itself functions as a structure that physically breaks off lipopolysaccharide (LPS) from the outer surface of neighboring bacteria, creating an osmolyte that is suspended in the swarm medium.^102^ Consistent with a role for LPS, *S. enterica* strains that are defective in the production of LPS are deficient in swarming.^153^ Ping *et al.* indicated that an influx-driving osmolyte should have a high molecular weight and small diffusion coefficient, and concluded that LPS is a reasonable candidate.^127^ Substances of low molecular weight such as salts or trehalose would be insufficient to cause the influx due to their relatively large diffusion coefficient. An alternative model for the activity of flagella physically removing LPS from the cell surface is that the production of flagella and LPS could be co-regulated. In *C. jejuni*, the production of LPS and flagella is linked by phosphoethanolamine transferase, an enzyme which modifies both the LPS anchor and flagellar rod protein.^154^ This is evidenced by mutants of this enzyme being defective in both LPS and flagella production.

Wu *et al.* proposed that metabolism byproducts associated with *E. coli* growth increase osmolarity in the swarm relative to the agar layer.^119^ Building upon this model, we suggest that the cellular activity associated with building and powering flagella could produce metabolites that increase osmolarity. In this model, the energetic requirements of flagellar activity increase metabolic turnover, the outcome of which is an increase in metabolites that function as osmolytes in the bacterial layer. Consistent with this, the energetic costs of building and rotating flagella can be a significant fraction of the entire cell budget.^155^ In *P. aeruginosa*, mutations that give rise to a greater rate of flagellar reversals or additional flagella give rise to hyperswarming.^81–85,156^ In the metabolite production model, the hyperswarmer phenotype could be attributed to the increased energetic requirements of reversing and rotating multiple flagella, which would increase metabolic turnover and the production of osmolytes that cause fluid influx from the agar layer.

The body of data presented in this section suggests that an important role for flagella in swarming is to increase the osmolyte concentration in the bacterial layer through the release of metabolites, LPS, or both. Data addressing this model in *P. aeruginosa* is lacking, which is a major challenge. Future studies should assess the extent to which osmolytes are increased by *P. aeruginosa* flagellar activity. Theoretical models could provide valuable insight here. For example, models that determine the osmolyte concentration and size required to produce fluid influx from the agar layer; measure osmolyte production due to metabolic turnover associated with powering flagella construction and rotation; or determine the concentration of LPS produced by flagella, could pinpoint the roles of flagella.

If the effect of flagella on swarming stems from increased liquid influx from the agar into a bacterial layer, it is unclear if such a mechanism would be considered sliding or swarming as defined by Henrichsen.^140^ On one hand, the volumetric expansion of the swarm would be due to liquid influx, which would suggest a sliding-like mechanism. The fluidic models proposed by Giverso *et al.*, Ping *et al.*, and Yang *et al.* are essentially volumetric expansion models that give rise to a sliding-like motility.^126–128^ On the other hand, the involvement of flagellar activity is consistent with Henrichsen's definition of swarming, though the details of how flagella cause expansion differ. Henrichsen described the role of flagella as facilitating the formation of aggregates or rafts that generate greater motile force rather than producing osmolyte. Nonetheless, these reasons suggest that *P. aeruginosa* swarms may need to be considered a combination of sliding and swarming mechanisms and that *P. aeruginosa* swarming motility may not strictly adhere to the Henrichsen definitions. As more mechanistic details about *P. aeruginosa* swarming are revealed, definitions of swarming may need to be refined or new surface motility classifications may need to be defined.

## DISCUSSION

We have reviewed biophysical factors that drive swarming in *P. aeruginosa*. Swarms have been largely identified by colony expansion and tendril formation, which are dependent on two critical factors: surfactant production and flagellar activity. Surfactant production is tightly regulated by QS, which ensures that swarming does not occur unless cells reach high density. This mechanism synchronizes swarming and enables the cells to move as a collective. Multiple models explain how swarm expansion and tendril formation are achieved once cells reach high density.

In particular, the studies convey that swarm expansion and tendril formation are coupled; processes that enable swarm expansion also facilitate tendril formation. In one model, the sensory detection of molecules guides tendril formation, which also results in swarm expansion. Fluid mechanics-based studies that consider swarms as thin films of liquid suggest alternative mechanisms. One model attributes swarm expansion and tendril formation to Marangoni forces that arise due to a surface tension gradient established by surfactants [Fig. 3(a)]. Another model attributes swarm expansion and tendril formation to pressure-driven flow due to the influx of fluid from the agar layer [Fig. 3(b)]. Here, fluid transport occurs due to the production of osmolytes in the bacterial layer, which would increase liquid flux from the agar layer into the bacterial layer. The incorporation of RLs yields a multilayer model in which pressure-driven flow gives rise to the volumetric expansion of the bacteria population on a surfactant layer [Fig. 3(c)].

Multiple models explain how osmolytes in the bacterial layer could be produced, including: through the direct action of flagella removing LPS from cell bodies, the co-regulation of LPS production with flagella, and metabolite production associated with growth and flagella operation. Moving forward, data showing a direct connection between flagellar activity and osmolyte production are necessary to establish a more comprehensive model of swarming in *P. aeruginosa*.

This review has covered biochemical mechanisms that give rise to swarming, including HAA and RL production, QS and RL sensing, and chemotaxis. In addition, it has covered biologically passive mechanisms that are based on fluid mechanics. To what extent is swarming due to an active biological system such as molecular sensing? To what extent is swarming explained by fluid mechanics? The answers are likely to involve an interplay between biological and fluid mechanics-based processes. Future models may need to take both aspects into account.

The bulk of swarming assessments has been performed using macromorphological measurements. A closer look at the single-cell dynamics or micromorphological features of swarms suggests that *P. aeruginosa* swarms do not adhere to early definitions of swarming given by Henrichsen and could involve mechanisms that are associated with both swarming and sliding.^140^ The requirement of surfactant production, the lack of cell aggregates, and the dense movement of cells at the swarm front suggest the involvement of a sliding-like mechanism. However, the strong dependence of colony expansion and tendril formation on flagella suggests a swarming-like mechanism. As more details of swarming are revealed, previous definitions may need to be refined or new classifications may need to be defined. As many studies will continue to use macromorphological assessments, quantitative metrics of surface motility have been proposed to improve classification across different conditions and strains.

### Environmental relevance of swarming

Swarming by *P. aeruginosa* has been observed in laboratory settings in which growth conditions are tightly controlled with respect to a number of factors including humidity, temperature, and agar content.^5,93,94,133^ Additionally, nutrient-limited conditions are required to induce swarming.^5,14,133^ *P. aeruginosa* inhabits diverse environments, including the human body, soil, plants, and waterways, and can sustain harsh conditions.^157–159^ The observation of swarming under tightly controlled conditions raises the question of to what extent is swarming relevant in such settings. At the fundamental level, swarming is a mechanism that facilitates the volumetric expansion of *P. aeruginosa* and its requirements are the production of RLs and flagellar activity. If these conditions are met, it is possible that volumetric expansion through swarming could occur in diverse environments. For example, *P. aeruginosa* can colonize the lung,^160–162^ which contains porous semi-solid surfaces and abundant fluids. This environment could promote surfactant production and flagellar activity, which could facilitate volumetric expansion. Consistent with this role, RLs and flagella-active *P. aeruginosa* have been isolated from patient lungs.^35,163,164^ Swarm patterns in diverse environments are likely to differ from those that are observed in lab settings. One particular aspect that requires consideration is that Petri dishes are two-dimensional environments. The dynamics of *P. aeruginosa* swarming in a three-dimensional environment, such as tissue, will need to be taken into account.

In natural environments, *P. aeruginosa* coexists with a multitude of microorganisms including *S. aureus.*^165–169^ These interactions can impact swarming behavior by disrupting the RL layer or by directly preventing *P. aeruginosa* growth.^42,170^ Therefore, understanding the interaction between *P. aeruginosa* swarm populations, surfactants produced by other species, and direct interactions with other bacterial species is essential to determine the role of swarming in natural and host settings.

### Closing the knowledge gap of swarming

Volumetric expansion on surfaces is a common bacterial process that is simply a mode of growth. While we have focused our review on *P. aeruginosa*, volumetric expansion through swarming is observed across multiple bacterial genera. Due to the large diversity of swarm phenotypes observed among different bacteria, it may be difficult to have a common definition of swarming that encompasses all species and swarm types. Focusing on swarming by *P. aeruginosa* has enabled us to disentangle the roles of QS, surfactant production, osmotic pressure, and flagellar activity. Moving forward, it will be important to determine how these systems cooperate to give rise to swarming.

## METHODS

### Strains and growth conditions

*P. aeruginosa* strains PA14^71^ and MPAO1^171^ were streaked from frozen stocks onto LB Broth-Miller (BD, Franklin Lakes, NJ) plates containing 2% Bacto agar (BD) and incubated overnight at 37 °C. Single colonies were inoculated into LB broth and incubated at 37 °C for 16 – 18 hrs in a roller drum at 20 rpm. Five microliters of overnight culture was inoculated onto M8 minimal medium containing 1 mM MgSO4, 0.2% glucose, 0.5% Casamino Acids (BD), and 0.5% Bacto agar (BD), and incubated at 37 °C as described previously.^172^ Timelapse images were acquired using IRIS as described previously.^42,43^

## Data Availability

The data that support the findings of this study are available within the article.
